# Benchmarking AutoML for regression tasks on small tabular data in materials design

**DOI:** 10.1038/s41598-022-23327-1

**Published:** 2022-11-11

**Authors:** Felix Conrad, Mauritz Mälzer, Michael Schwarzenberger, Hajo Wiemer, Steffen Ihlenfeldt

**Affiliations:** Technical University Dresden, Faculty of Mechanical Science and Engineering, 01062 Dresden, Germany

**Keywords:** Computational methods, Computational science, Scientific data

## Abstract

Machine Learning has become more important for materials engineering in the last decade. Globally, automated machine learning (AutoML) is growing in popularity with the increasing demand for data analysis solutions. Yet, it is not frequently used for small tabular data. Comparisons and benchmarks already exist to assess the qualities of AutoML tools in general, but none of them elaborates on the surrounding conditions of materials engineers working with experimental data: small datasets with less than 1000 samples. This benchmark addresses these conditions and draws special attention to the overall competitiveness with manual data analysis. Four representative AutoML frameworks are used to evaluate twelve domain-specific datasets to provide orientation on the promises of AutoML in the field of materials engineering. Performance, robustness and usability are discussed in particular. The results lead to two main conclusions: First, AutoML is highly competitive with manual model optimization, even with little training time. Second, the data sampling for train and test data is of crucial importance for reliable results.

## Introduction

Machine Learning (ML) is applied in materials science for materials property analysis, the discovery of new materials or quantum chemistry^1^. However, the application of ML remains a time-consuming effort. Moreover, the constant advancement of new ML models makes it difficult to keep up with the latest developments. A solution to these issues could be automated machine learning (AutoML), which simplifies the ML modeling process for various application domains, including healthcare, engineering or education^2^. AutoML provides a chance to open up ML to non-experts, improving the efficiency of ML and accelerating the research with ML. Overall, AutoML is increasingly applied by data scientists. A Kaggle survey from 2021 states that about 58% of data scientists use AutoML frameworks, trending upwards^3^. In addition, data scientists who actively use AutoML encourage more widespread application of automated techniques in ML^2^. The growing adaptation also widens the scope of related fields for AutoML. In this respect, explainable artificial intelligence (XAI) is of rising interest to help users to interpret the models and results generated by AutoML. However, automation of the data processing pipeline in materials engineering is applied only at low automation levels in the definition of Karmaker et al.^4^. Automation is encountered most frequently for hyperparameter optimization^5,6^ or neural architecture search^7^. The automation of data preprocessing, feature engineering or model explainability is hardly covered until now. Looking at experimental materials design in particular, a reason for the low spread of AutoML might be caused by frequently occurring data characteristics. The benefits and drawbacks of AutoML have been generally discussed in multiple benchmarks and reviews, yet, a practical analysis focusing on materials science and its often very small tabular datasets is not publicly known.

**Figure 1:**
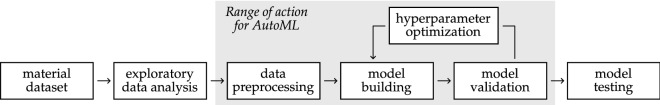
Data processing pipeline for a typical data mining workflow in materials design, starting with a material dataset and ending with model testing. Steps within the highlighted space can be automated by use of AutoML tools.

Two points of view are relevant for the following considerations: a domain specific perspective (“What problems can be solved with ML in materials design?”) and a technical perspective (“What behavior do AutoML frameworks show for small datasets?”). Several recent reviews proposed the use of machine learning for the prediction of mechanic, electrical, thermodynamic and other material properties^8–13^. These studies show the specifics and challenges of applying ML in the materials domain. Available datasets are often very small because the data points are based on costly experiments. Common features specify material composition and processing parameters, and feature types may be numeric or categorical. AutoML has emerged as a simplifying solution for repetitive optimization loops in the increasingly complex modeling workflow, displayed in Fig. 1. Hyperparameter optimization (HPO) was the starting point and still is the topic at the core of AutoML. It culminated in ways to deal with what Hutter et al.^14^ refer to as *combined algorithm selection and hyperparameter optimization* (CASH) problem. There are multiple approaches to solving the CASH problem, Bayesian optimization, genetic programming or heuristics based ones. Until now, AutoML has been extended to also include meta-learning^15^, neural architecture search^16^ and data preparation^17–19^ in order to cover more steps of the data processing pipeline by a single tool. The highlighted parts of the pipeline in Fig. 1 can be automated and hence be treated as a single step in the workflow of materials researchers. Ideally, AutoML reduces time and effort as well as increases robustness and reliability throughout the data mining process.

The state of the art on the capabilities of individual AutoML frameworks can be taken from recent surveys and benchmarking studies: He et al. provided a thorough discussion in 2021^20^. They identify eight open challenges, in sync with earlier reviews^21,22^. The challenges consist of search space flexibility, a broadening of application tasks, interpretability, reproducibility, robustness, joined hyperparameter and architecture optimization and learning transferability. Elshawi et al.^23^ additionally make note of a need for higher user-friendliness, to gain widespread acceptance. Robustness, especially the facet that He et al. refer to as a *higher modeling resilience* in real datasets, plays a vital role in this study. In the following, it is intended to contribute to a better understanding of robustness in a domain-constrained manner. A noteworthy approach towards more clarity for AutoML robustness is taken by Halvari et al.^24^. They set up an AutoML benchmark with different intentionally introduced data corruptions and observe training behavior as well as individual sensitivities for the resulting pipelines. A broader and more general evaluation was given by Truong et al.^25^ containing several AutoML tools and nearly 300 datasets. They grouped multiple research questions into segments and discussed the framework behavior for open source and commercial tools. Truong et al. use fixed train-test splits and divide datasets with sample sizes above and below 10,000 samples. Gijsbers^26^ provided an open-source framework for benchmarking various AutoML Frameworks, which is used to compare 39 datasets. A comparison between AutoML frameworks and manual data analysis is evaluated by Hanussek et al.^27^. They encourage the use of AutoML for general purposes, showing that human performance is in most cases matched if not surpassed. Although there are detailed framework comparisons, a focus on small datasets with sample sizes between 10 and 10,000 data points is still missing. Also missing are domain-specific evaluations in general, let alone evaluations in materials engineering. Lastly, thorough discussions of human evaluations as opposed to AutoML based ones are rare.

This contribution addresses three identified gaps. AutoML is evaluated with respect to small dataset sizes, the constrained domain and traditional approaches. A variety of datasets from materials engineering is analyzed by four different AutoML frameworks. Each of the datasets is related to at least one scientific publication including a manual ML analysis, and most contain less than 1000 datapoints. Observation shows that AutoML is applicable and simplifies ML application in materials science. Thereby, AutoML often improves or achieves almost the same performance of top state-of-the-art ML applications. The combination of AutoML frameworks proposed in this study can further enhance performance. As a substantial contribution, it is shown that robustness and trustworthiness of ML models is improved through nested cross-validation (NCV). The implementation of the study is provided alongside this publication. It allows to use the four AutoML frameworks in combination in an easily extensible way.

The next chapters describe the details in the following order: First, the choice of datasets and AutoML frameworks is introduced. Second, the implementation strategy is explained before third, a performance comparison for the AutoML frameworks is presented. Fourth, the observations and their implications are discussed, and lastly, conclusions and future prospects are presented. Framework details and information about data and code availability are appended.

## Results

To begin with, the datasets that will be used in the remainder of this paper are presented in detail. This is followed by an explanation of the selection process for the AutoML frameworks. NCV and the unified data preparation are introduced before the actual benchmarking comparison is presented.

### Materials engineering datasets

The criteria for the selection of the datasets were the availability in tabular format, the existence of at least one publication showing a manual data-mining investigation, the formability as a regression task and the affiliation to the domain of materials design. The restriction to tabular data is chosen as this is necessary for applying the AutoML frameworks without manual intervention. However, it is important to mention that certain tasks in materials engineering (e.g. the majority of MatBench datasets^28^) need featurization of the chemical composition as preprocessing to get a tabular dataset. So far, this preprocessing step has not been implemented in any AutoML framework. For this reason, datasets that require featurization have not been considered. The restriction to focus only the highlighted steps in Fig. 1 allows to evaluate the possibilities and limitations of what can already be fully automated. Regression tasks are predominantly present in the domain of materials design^29^, which is why an additional restriction was made to just those. Transferability of the results to classification tasks is expected due to high similarity of the algorithms. Unsupervised learning is out of scope for this study and not further addressed as it would imply an inherently different evaluation workflow. Twelve datasets from recent literature as well as public data repositories match these criteria.

As seen in Table 1, the selected datasets vary with respect to the sample and feature size and have up to six target properties. They contain experimental data exclusively, which limits the size of the datasets. Only Yin-2021 contains a few simulated data points. The datasets were grouped into three categories to emphasize different sample sizes. *Very small* datasets contain less than 200 samples, *small* datasets more than 200 and *large* with significantly more than 1000. The threshold was chosen following Zhang et al.^8^, so that the scaled error (mean absolute error scaled by the value range) is more than 10% for the very small datasets, between 5 and 10% for the small datasets and less than 5% for the large datasets. Codenames were given to the datasets in order to allow easier referencing. The naming scheme follows the rule “first author dash publication year”, with UCI-concrete and Matbench-steels being exceptions given their widespread acceptance under these names.

The selected datasets stem from three different material categories: concrete, metal and fibre reinforced polymers (FRP). The dataset UCI-concrete, assembled by Yeh^30^, stands out as it has been published in the University of California (UCI) machine learning repository since 2007. It was discussed in over 20 publications regarding the use of ML for prediction of material properties. Table 2 provides information on the most important publications in this regard. UCI-concrete is renown for being one of the main reference models for tabular data in materials engineering. The features in UCI-concrete are all numerical, representing the composition and age of concrete. Other datasets describing composition, age and material properties of concrete aggregates are Atici-2011, Bachir-2018, Huang-2021 and Koya-2021. For Atici-2011^31^ only the data for ’Model 1’ was fully available and used in this study. Bachir-2018^32^ is a dataset investigating the use of rubber as an aggregate in concrete. Huang-2021^33^ analyzes the mechanical properties of carbon nanotube reinforced concrete. It contains categorical features that are already encoded. In Koya-2021^34^ the data-mining results from the dataset provided in the mechanistic-empirical pavement design guide from the American Association of State Highway and Transportation Officials^35^ are presented.

Datasets containing the chemical composition and information about the material manufacturing process from the metal domain are Guo-2019 and Hu-2021. Guo-2019^36^ is by far the largest dataset in this study, describing steels from industrial environments. Hu-2021^37^ considers aluminum alloys. Matbench-steels^28^ considers only the composition of steel alloys, the dataset was first published by Bajaj et al.^38^. The results and the dataset version from the materials benchmark by Dunn et al.^28^ are used in this study. Another dataset from the metal category is Xiong-2014^39^. It describes the relationship between the process parameters in gas metal arc welding and the single weld bead geometry for usage in additive manufacturing (AM).

The scope of the Yin-2021^40^ dataset is to characterize the interfacial properties of fibre reinforced composites based on the material properties of the components and test conditions. The dataset Su-2020^41^ is used for the investigation of the interfacial bond strength between concrete and FRP. Two different experiments were conducted, thus a distinction as Su-2020-1 and Su-2020-2 is included. Su-2020-1 analyzes the bond of FRP on plane concrete and Su-2020-2 analyzes FRP on concrete with a groove.

**Table 1:** Overview of investigated datasets ordered by size and their properties in the published version, the large, small and very small datasets are divided by the dashed line categorical features (cat.), interfacial shear strength (IFSS), coefficient of thermal expansion (CTE).

Alias + Source	Domain	Targets	Size	Features	Cat.	HP. Tuning	Train-Test-Split	$R^2$	RMSE
Guo-2019^36^	Steel	tensile strength, yield strength, elongation	63162	27	✗	None	5-fold CV	$\checkmark$	$\checkmark$
UCI-concrete^30^	Concrete	compressive strength	1030	8	✗	See Table 2	See Table 2	$\checkmark$	$\checkmark$
Yin-2021^40^	FRP	IFSS, pullout force	900	11	✗	Manual	89/11	$\checkmark$	✗
Hu-2021^37^	Aluminum	tensile strength, yield strength, elongation	896, 860, 783	27	$\checkmark$	GS	80/20	$\checkmark$	$\checkmark$
Matbench-steels^28^	Steel	yield strength	312	14	✗	Automatminer	5-fold NCV *	✗	✗
Atici-2011^31^	Concrete	compressive strength	140	3	✗	Manual	85/15	$\checkmark$	$\checkmark$
Su-2020-2^41^	FRP + concrete	bond force-2	136	5	✗	RS+GS	80/20	$\checkmark$	$\checkmark$
Su-2020-1^41^	FRP + concrete	bond force-1	122	7	✗	RS+GS	80/20	$\checkmark$	$\checkmark$
Huang-2021^33^	Concrete	compressive strength, flexural strength	114	13	✗	Manual	80/20	$\checkmark$	✗
Bachir-2018^32^	Concrete	compressive strength	112	3	✗	Manual	85/15	$\checkmark$	$\checkmark$
Koya-2018^35^	Concrete	compressive strength, Young's modulus, modulus of rupture, split tensile strength, Poision's ratio, CTE	110	10	$\checkmark$	Manual	10-fold CV	$\checkmark$	$\checkmark$
Xiong-2014^39^	AM of steel	width, height	43	4	✗	Manual	72/28 *	✗	✗

*Exact train-test-split available.

**Figure 2:**
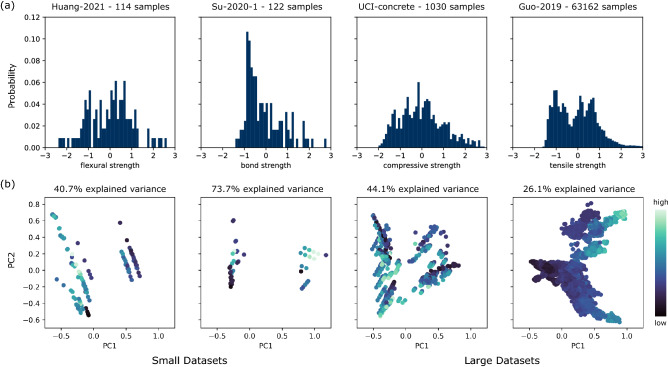
Design space visualisation from chosen datasets. (**a**) Histogram of one target value normalized via standard scaler. (**b**) Representation of the standardized input feature space for the selected datasets via visualization of the first two principal components , the color of the points represents the target value.

The datasets show significant differences in the value distribution within the feature space and target variables even after normalization. A principal component analysis (PCA) visually highlights the distribution differences. A selection of dataset visualizations is displayed in Fig. 2 to represent the conclusions across all datasets. For the two rightmost larger datasets, the histograms in Fig. 2a depict a smoother distribution of the target variable. However, the targets are not normally distributed even for the largest dataset Guo-2019, which shows three modes. For the smaller datasets, the data is more sparse. Su-2020-1 shows a significant concentration and some outliers. Fig. 2b shows the value distribution of the first two principal components (PCs) for the full dataset. To emphasize possible clusters regarding the target variable, the true label (target variable) is encoded in the color of the sample points. Additionally, the explained variance from the first two principal components is given as an annotation in Fig. 2b. In summary, large datasets are more centered and more evenly distributed than small and very small datasets, also showing less outliers.

In all but one studies, hyperparameter optimization (HPO) was used to improve data modeling. The techniques applied were not disclosed (manual HPO), grid search (GS) or random search (RS). The same observation can be made for studies related to UCI-concrete, for which only two publications hinted at a different HPO technique, cf. Table 2. The result of Feng et al.^42^ is used as reference performance further on since it is the best literature result obtained via cross-validation (CV). A noteworthy observation is that recent studies applied feature engineering before the modeling process. Han et al.^43^ constructed several additional features by dividing two of the given input features. Furthermore, Chakraborty et al.^44^ performed a feature selection via recursive feature elimination (RFE).

**Table 2:** Overview of the literature using the UCI-concrete dataset. SVM (Support Vector Machine), GBT (Gradient Boosted Trees), BNN (Bagged NN), GBNN (Gradient Boosted NN), WBNN (Wavelet Bagged NN), WGBNN (Wavelet Gradient Boosted NN), Regression Forest (RF), Decision tree (DT), Gridsearch (GS), Gridsearch + Feature construction (GS+).

Source	Year	Model	Param. Tuning	Train-Test-Split	$R^2$	RMSE
Yeh^30^	1998	Linear Regression	Manual	75/25	0.770	N/A
Yeh	1998	Neural Network (NN)	Manual	75/25	0.914	N/A
Chou^45^	2011	SVM	Manual	10-fold CV − avg	0.8858	5.619
Chou	2011	GBT	Manual	10-fold CV − avg	0.9108	4.949
Erdal^46^	2013	BNN	GS	90/10	0.9278	4.870
Erdal	2013	GBNN	GS	90/10	0.9270	5.240
Erdal	2013	WGBNN	GS	90/10	0.9528	5.750
Golafshani^47^	2019	Symbolic Regression	Manual	75/25	0.8008	10.6984
Han^43^	2019	RF + 1 const. Feat.	GS+	$50 \times 90$/10 split − avg	0.9322	4.434
Nguyen-Sy^48^	2020	GBT [XGBoost]	GS	10-fold CV − avg	0.93	4.270
Feng^42^	2020	AdaBoost with DT	GS	10-fold CV − avg	0.952	4.856
Chakraborty^44^	2021	GBT [XGBoost]	GS + RFE	90/10	0.979	2.650

### AutoML frameworks

Tools used in similar benchmarks^21,24–27^ as well as those mentioned in a low-code AutoML review from 2021^49^ were accumulated to an initial set of candidates for use in this study. Several selection criteria were applied to identify representative candidates. Two task independent design decisions were applied at first: Commercial and proprietary tools were excluded to ensure accessibility and transparency. Not actively maintained frameworks (without substantial commits or releases within the last year) were excluded to ensure actuality. Additional criteria were derived from the tasks used in this study. The two following exclusions are justified by the exclusive use of regression tasks based on small tabular data in this benchmark: Tools targeted at unrelated ML tasks (e.g., image segmentation or reinforcement learning) were excluded to ensure applicability. Tools with a focus on neural networks and neural architecture search (NAS) were excluded due to the required data size. Two of the remaining frameworks, H2O and TPOT, were used in all five, Auto-sklearn in four of the above mentioned benchmarks. These were included. Auto-sklearn^15^ is among the first academic tools that is still actively maintained. H2O^19^ is of interest due to the possibility to run on distributed machines. TPOT^17^ differs from the others by using a genetic algorithm for HPO. Additional promising candidates were MLjar, AutoGluon, Polyaxon and PyCaret. Each of these can have individual benefits depending on the area of operation (e.g. cloud-integration, low-code focus or permissive licensing), which is out of scope for this research. MLjar^18^ was added to the selection due to a particularly intuitive ML explainability function as well as supposedly good performance in a (not scientifically reviewed) benchmark^50^.

With the analysis of feature importance, all frameworks offer a minimal entry towards XAI. H2O and MLjar offer extended functionality with SHAP values and corresponding plots for further analysis (“Advanced” XAI). Information about the used frameworks beyond the condensed comparison in Table 3 is given in the “Methods” section.

**Table 3:**
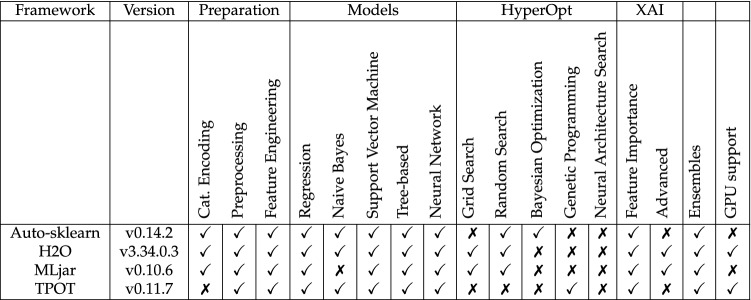
Features of the applied AutoML framework.

### Implementation and experimental setup

Several procedures regarding the data and its processing were fixed to ensure comparability and reproducibility in this study. First, the datasets were extracted from their original publications. Some invertible modifications have been made to use SI units and resolve mixed fractions into floating-point numbers. All datasets were saved as CSV files with separate text file descriptors pointing to the input and output vector columns. No further data preparation or feature engineering was applied. These tasks were deliberately given to the AutoML frameworks as part of automated data preprocessing. A manual data modification was only necessary for the Hu-2021. In this case, multiple categories were assigned to a single categorical feature. Common encoding strategies would fail otherwise (e.g., one-hot encoding). Second, the datasets were handed to an automated training routine to train the AutoML models (see “Methods” section for a detailed description of the training routine). For all AutoML frameworks, the training metric was set to $\mathrm {R}^2$. All other training parameters were set to default values. Third, the training results were verified and compared to the results from the original publications.

**Figure 3:**
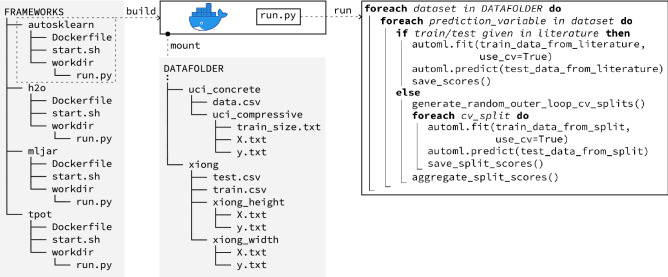
Workflow for the evaluation process.

All datasets were transformed into single output regression problems, referred to as *task*. Datasets with multiple prediction values were trained with separate models for each prediction variable (task). Only input features from the corresponding literature were used. Modified codenames were given to the tasks in analogy to the abbreviations for the datasets. The naming scheme follows the rule “first author underscore prediction variable”. The choice of train and test splits can heavily impact the prediction result, especially for small datasets. Identical sample allocations in train and test data are used to compare literature and AutoML results, if available. However, as shown in Table 1, not all studies provide the exact sample allocation. The problem was circumvented by the implementation of an outer loop via NCV with five folds. For each fold, the train data is used for the combined algorithm selection and hyperparameter optimization via CV. The test data from the outer loop is used for the performance evaluation. The train-test proportion in the outer loop is adapted to match the settings from the original publications. In this way, the problem of unknown sample allocations is addressed by repeating the experiment five times with different train-test splits in the outer loop. The aggregated results can be used for an estimate of the true predictability. The number of inner folds is set to 10 for all experiments. Compared to the simple CV, where the outer loop is omitted and the evaluation is performed on the inner splits, any bias through possible data leakage can be eliminated with the NCV^51,52^. Additionally, the NCV is an appropriate means to avoid overfitting and to evaluate the overfitting effect of the HPO^53^ even though it is more computationally expensive. The outer split was initialized with *sklearn.ShuffleSplit(seed = 1)* for all frameworks.

In terms of hardware, all experiments were conducted on virtual machines, using 8 isolated CPU-cores (Intel(R) Xeon(R) Gold 6136 CPU @ 3.00GHz). The machines had access to 20GB RAM, with RAM not being a bottleneck parameter. A macroscopic view on the implementation routine is depicted in Fig. 3. Each framework had exclusive access to the hardware during the experiments of 15 and 60 min of training time per dataset and cross-validation split.

However, as of Auto-sklearn v0.14.2 an implementation bug was present that required manual reset of the framework between runs to free memory. Further noteworthy is that all but one dataset contain only numerical inputs. Only Koya-2018 contains categorical inputs. As TPOT cannot handle categorical inputs, it is removed from the comparison for the Koya-2018 dataset.

### Performance comparison

As with the sample allocations, identical performance measures in the related literature is used for the comparisons. Hence, the results are evaluated with $\mathrm {R}^2$ and root mean square error (RMSE) for most datasets. Matbench-steels was evaluated with the mean absolute error (MAE) and the Xiong tasks with the mean absolute percentage error (MAPE). A normalized expression is used further on to establish comparability. The performance scores for comparison across datasets are expressed as relation between the AutoML and best literature score, given by

1 $$\begin{aligned}&\mathrm {R}^2_{rel} = \frac{\mathrm {R}^{2}_{automl}}{\mathrm {R}^2_{literature}}, \end{aligned}$$

2 $$\begin{aligned}&\mathrm {RMSE}_{rel} = \frac{\mathrm {RMSE}_{literature}}{\mathrm {RMSE}_{automl}}, \end{aligned}$$

3 $$\begin{aligned}&\mathrm {MAPE}_{rel} = \frac{\mathrm {MAPE}_{literature}}{\mathrm {MAPE}_{automl}}, \end{aligned}$$

4 $$\begin{aligned}&\mathrm {MAE}_{rel} = \frac{\mathrm {MAE}_{literature}}{\mathrm {MAE}_{automl}}. \end{aligned}$$

Achieving the same performance as the literature will lead to a relative score of 1. Better performance yields a relative score above 1 and worse below 1, for all performance measures.

Four tasks from Koya (coefficient of thermal expansion, Poisson’s ratio, Young's modulus, split tensile strength) were neglected in the evaluation because no valuable model ($R^2 > 0.10$) could be found in the literature or by any of the frameworks. Thus, neither the literature nor the AutoML frameworks could find a relationship between the features and the labels, indicating that this relationship is not mapped in the dataset.

**Figure 4:**
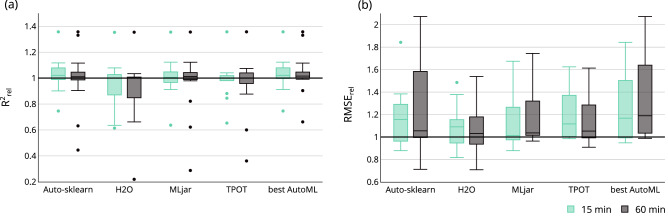
The mean relative scores of the four tested AutoML frameworks and the *best AutoML* aggregation per training time. (**a**) Mean relative score based on $\mathrm {R}^2$ (**b**) Mean relative score based on RMSE.

The results from the single prediction tasks are summarized for an overall comparison between the AutoML frameworks and literature results. The outer CV results are aggregated into a boxplot for each framework, their mean relative scores for every task is shown in Fig. 4. An ensembled result is shown next to the results of the individual frameworks. This represents the AutoML framework with the highest mean relative score for every task.

**Figure 5:**
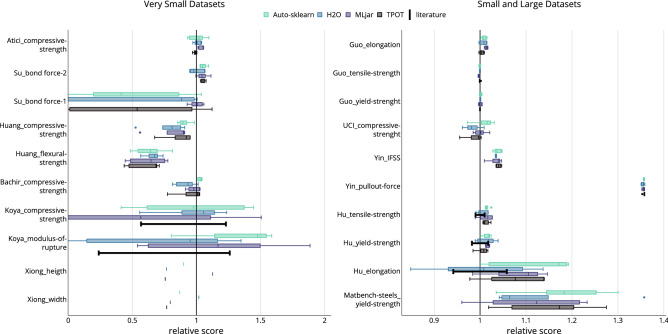
The relative score from the outer splits per task. Relative score means $\mathrm {MAE}_{rel}$ for Matbench-steels, $\mathrm {MAPE}_{rel}$ for Xiong and $\mathrm {R}^2_{rel}$ otherwise. For Hu and Koya the literature provides a performance range, represented by a black “error bar”.

In general, the difference between the AutoML frameworks is within a relatively small margin. Auto-sklearn and MLjar have a median of the mean $\mathrm {R}^2_{rel} > 1$ for both training times, H2O achieves this only for the 60 min training time setting (Fig. 4a). Thereby, Auto-sklearn achieves the best average result, with its median $\mathrm {R}^2_{rel} = 1.02$ for a runtime of 15 min. MLjar follows with its median $\mathrm {R}^2_{rel} = 1.01$ for a runtime of 60 min. TPOT, however, achieved a median $\mathrm {R}^2_{rel} < 1$ for either time setting, but only by a small margin. It is to mention that the longer training time of 60 min does not ultimately lead to better performances in this experiment. The results vary within a wide range. Each framework may achieve a mean relative score of up to 1.36, all for the task Yin_pullout_force (see Fig. 5). Apart from that, some tasks perform considerably worse than the literature reference with a mean relative score below 0.6.

For RMSE$_{rel}$, all four AutoML frameworks surpassed the literature results for 15 min as well as 60 min training time (Fig. 4b). Auto-sklearn achieved the best result with its median $\mathrm {RMSE}_{rel} = 1.16$, followed by TPOT with a median of $\mathrm {RMSE}_{rel} = 1.12$, both with 15 min training time The aggregation over all frameworks *best AutoML* achieved a median of $\mathrm {RMSE}_{rel} = 1.19$ for 60 min training time, outperforming all tasks compared to the literature except Gou_tensile-strength. The performance gain of the *best AutoM*L compared to the individual frameworks is significantly greater for the relative RMSE than for the relative $\mathrm {R}^2$. Again, the longer training time does not appear to enhance performance of the single frameworks and the results vary within a wide range.

### Dataset specific results

The results for the included tasks are shown in Fig. 5. The x-axis is cut at $\mathrm{relative\:score} = 0$ = 0 for better visualization. The results not shown indicate a negative $\mathrm {R}^2$ and imply that no valuable model was found.

The results for the very small datasets vary the most over the outer splits from the NCV. The large datasets show the least variation. For the small datasets, the results are still mostly consistent. However, Hu_elongation and Matbench-steels_yield-strength are an exception, which was for Hu_elongation also detected by Hu et al.^37^. Hu_elongation and Matbench-steels_yield-strength have a spread of the relative score of 0.2 on average and are the only tasks from the small datasets with a higher spread, compared to task Atici_compressive-strength with the smallest spread of 0.1 on average from the very small datasets. The results for both tasks from Koya-2018 exhibit the largest variation overall with an average of 1.3 for Koya_modulus-of-rupture and an average of 1.8 for Koya_compressive-strength. Again, Koya et al.^34^ identified this behavior in their own research. No varying performance is presented for Xiong-2014 as no NCV was applied. Xiong et al.^39^ provide the exact train test split, allowing the comparison of AutoML and manual data-mining on an identical train test split, cf. Fig. 3.

Considering the comparison of AutoML with literature results, AutoML outperforms the manual approaches for large and small datasets. At least two AutoML frameworks achieve better median results than the literature for all tasks except Gou_tensile-strength, for which the relative scores are very close to 1. Auto-sklearn was able to outperform the literature in every task of the large and small datasets, except for Gou_tensile-strength. MLjar outperformed all of these tasks except Gou_tensile-strength and Gou_yield-strength. The performance for very small datasets varies. The AutoML frameworks did not achieve better median results than the literature consistently. As for the global comparison, the AutoML frameworks match closely in performance on the dataset level. No framework had a higher score in all runs than the others. The manual approach was not surpassed by any of the AutoML frameworks in three tasks.

**Figure 6:**
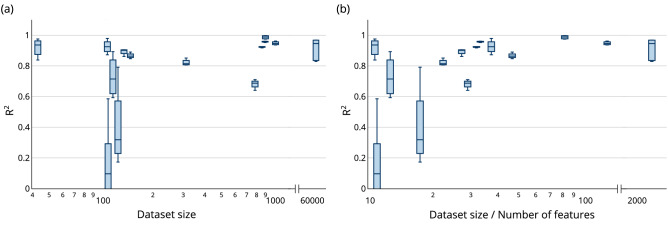
The performance $\mathrm {R}^2$ of with respect to the dataset size and shape, one box represents all outer loop runs of one dataset. (**a**) $\mathrm {R}^2$ over dataset size, the boxes are slightly shifted to avoid overlapping, without affecting the interpretation of the graphic. (**b**) $\mathrm {R}^2$ over dataset size divided by number of features.

The datasets examined show that a large ratio between data set size and number of features (shape ratio) generally leads to a better prediction performance, cf. Fig. 6b. However, from a shape ratio above 50, there are no longer any significant improvements. Prediction tasks with a shape ratio below 30 were prone to a low performance, as all predictions in this study with a $\mathrm {R}^2<0.8$ come from those tasks. They also have a larger spread in the results, meaning that they are more influenced by the specific train-test splits. These are not strict relationships but rather general tendencies. The shape ratio is a better measure than pure data set size in this aspect, as the trend is less clear when focusing only on data set size, see Fig. 6a.

## Discussion

The following discussion evaluates several implications of the results. Special attention is drawn to the spread of the results. Further aspects are training time, usability and algorithm choices.

### Data split dependency

Some tasks in this investigation showed a heavily varying performance because of the different random train-test split in the outer loop of the NCV. This can be seen in the results for the very small datasets, Hu_elongation and Matbench-steels_yield-strength. For example, in the task Su_bond-1, the performance of H2O for a runtime of 60 min varies between $\mathrm {R}^2_{rel} = 1.01$ and $\mathrm {R}^2_{rel} = -1.66$ for the nested cross-validation. This variation strongly influences the interpretation of the obtained results. For a single train-test split, the evaluated performance can under- or overestimate the true performance of the model. Furthermore, no information can be gained over the performance stability for different random train-test splits. For the cross-validation, it is necessary to provide information about the mean performance and the deviation. Only then it is possible to get information about the stability of the performance.

For tasks with a high variance in the performance of differently chosen train-test splits, the training dataset might not capture enough of the available information. Therefore, the trained models are susceptible to overfitting and underfitting, as seen in Yin_pullout-force, or, respectively, do not develop generalizability. This problem is more likely to occur with small data sets because the sample size used for the train or test split may not adequately represent the underlying distribution, indicated by Fig. 6. There have been many scientific contributions describing the observed high prediction error as a consequence of the used sampling strategy: Some researchers state that this behavior is to be expected^54^ and show that the influence of the sampling will decrease with an increasing dataset size^52,55^. Others discuss mitigation techniques, for example non-parametric sampling^56^ or bootstrap variants^57^. Even though it is a known problem, it is seldom addressed. For the very small datasets only Koya et al.^34^ evaluated this aspect. Reason for this lack of popularity might be given in the hidden availability in data analysis tools such as scikit-learn (only ordered and random sampling is available in convenience function sklearn.model_selection.train_test_split^58^). Therefore, a thorough comparison between AutoML and the manual approach is impossible in this context. However, the observations suggest a cautious application of ML and hence AutoML to small and very small data sets. For this, careful selection of training and test data is essential. In particular, applying nested cross-validation increases the trustworthiness of the results.

### AutoML vs. literature

The higher mean in the relative $\mathrm {R}^2$ scores of Auto-sklearn, MLjar and H2O for a runtime of 60 min plus Auto-sklearn and MLjar for 15 min show the potential of modern AutoML frameworks. The use of AutoML results in higher mean performance than the manual data mining process. The relative scores of the single AutoML frameworks compared to the literature vary a lot between the single tasks. This is either due to the fact that the manual data mining process could not obtain an optimal task-specific result or due to dataset-specific weaknesses of the AutoML frameworks. Another point that can encourage this behavior is the dependency of the performance on the train-test split, which is often not considered in the literature. One main reason for AutoML frameworks performing so well is their broad model and hyperparameter space and the efficient techniques for optimizing in this large space. Combining the AutoML frameworks (*best AutoML*) can further enhance the relative scores since the model and hyperparameter space are even larger than in the individual frameworks. For the mean relative RMSE scores the advantage of the AutoML frameworks is even greater than for $R^2$, which indicate fewer outliers in the AutoML models, as the RMSE is more sensitive to outliers compared to $R^2$. The observation of the median relative scores leads to the same conclusions as shown here.

AutoML frameworks surpassed most results from the literature. Only Huang_flexural-strength, Huang_compressive-strength and Gou_tensile-strength were not matched. Huang-2021 is the only dataset where the regression of multiple outputs is faced with a multi output approach in the literature. Accordingly, a transformation to a single output problem was not applied. Multi-output approaches can surpass the performance of their corresponding single output approaches, which is shown by Ma et al.^59^ and Kuenneth et al.^60^. Relations between the targets can provide useful prediction-related information, which is completely ignored with the single task method. Using a multi output approach is impossible with the investigated AutoML frameworks since each output has to be formulated as a separate task. In contrast, the AutoML frameworks greatly outperformed the literature result for the task Yin_pullout-force. This is particularly remarkable as the model selection (gradient boosting regressor) did not differ significantly. Yin and Liew^40^ describe a significant overfitting effect in their studies, which is less present in the combined AutoML and NCV approach.

There is no clear trend for any of the literature HPO methods affecting the results. In most of the literature, no further details on HPO are presented (manual methods), so the actual effort remains unclear. The AutoML frameworks could outperform all literature results that use GS, RS or both. Special attention is to draw at Gou_tensile-strength. Here, no HPO was applied in the literature, and the default values of a random forest were used. However, no AutoML framework achieved better median results in this task. In contrast, the performance of Auto-sklearn and MLjar for the UCI_compressive-strength task should be highlighted. Although many researchers analyzed this dataset with various models, HPO and feature engineering techniques, these two AutoML frameworks outperformed the best literature result obtained with cross-validation by Feng et al.^42^.

### Framework comparison

Comparing the four AutoML frameworks shows that Auto-sklearn is the best performing AutoML framework overall, closely followed by MLjar. Particularly remarkable is the unmatched performance of Auto-sklearn for the 15 min runtimes. The worse performance of Auto-sklearn with 60 min runtimes indicates an overfitting effect. The warm start method used by Auto-sklearn promotes its strong performance in the short training time. This advantage disappears for the training time of 60 min, there MLjar can surpass the results from Auto-sklearn. The results of MLjar are the most robust in this study, having the smallest gap between quantiles. Nevertheless, no AutoML framework was able to outperform the others consistently. Furthermore, every AutoML framework was the best performing framework for at least two tasks in the 60 min runtime and at least one task in the 15 min runtime. Each AutoML framework investigates a different model and HP space and follows a different approach to solve the CASH problem. Hence, the performance of the AutoML frameworks differs depending on the task and data. As a result, for achieving the best performance for a given data-mining problem, it is beneficial to run several AutoML frameworks compared to just using one framework. This performance gain can be seen in Fig. 4, with the superior performance of the best AutoML *best AutoML* aggregation in comparison to the single frameworks.

### Training time

The extended training time of 60 min does not lead to a significantly increased performance of the single AutoML frameworks compared to the training time of 15 min. For Auto-sklearn, the median relative $\mathrm {R}^2$ and relative RMSE are even higher for the short training time. The performance decrease is related to overfitting induced by the HPO. Consequently, runtime needs to be treated as a new hyperparameter. Furthermore, the necessity of the nested cross-validation is strengthened. A similar overfitting is also visible in the other AutoML framworks. However, H2O and especially MLjar show some improvements for a longer training time, which is justified by a finer search using Grid Search or Random Search as HPO. Nevertheless, a further extended training time is not expected to improve the results for the single AutoML frameworks. In contrast, the *best AutoML* aggregation showed an increased performance of the median relative RMSE and consistent performance of the median relative $\mathrm {R}^2$. For the relative RMSE, although no single framework was able to increase the performance, the aggregation showed the benefit of this approach. The performance only decreases if none of the frameworks achieves the best performance of 15 min training time, and increases as soon as one of them can surpass it. Nevertheless, due to the general trend of the individual frameworks, a significant performance improvement is not to be expected with a further increased training time for *best AutoML*.

### Model selection

Despite having similar performances for the presented datasets and their tasks, the AutoML frameworks identify different types of models as best performing. Auto-sklearn and TPOT, both built upon the sklearn library, found gradient boosting regressors as the best performing model in most cases. Other often chosen models for TPOT were also tree-based, in particular XGBoost, extremely randomized trees and random forests. For Auto-sklearn the follow-up models were not tree-based: stochastic gradient descent (SGD) linear regressor, support vector machine (SVM), Gaussian process regression and Bayesian automatic relevance determination (ARD) regression. MLjar found almost exclusively tree-based models as the best performing models. Extremely randomized trees was the most frequent, followed by Catboost, XGboost, random forest and LightGBM. In contrast, H2O found neural networks as best performing ones. Relevant choices were also tree-based, namely gradient boosting machine, XGBoost, random forest and extremely randomized trees. The tree-based models show more robust results, with less hyperparameter sensitive spread. Overall, tree-based ML models were most frequently evaluated as the best performing models. These were followed by neural networks (H2O only) and linear regressors.

### Usability

The usability of the AutoML frameworks is similar and comparable to classic ML frameworks such as sklearn or XGBoost. In all four cases the API is well documented. Problems can arise with mutually exclusive dependencies, hence isolations are necessary for comparison. All frameworks run on multiple operating systems (OS), only Auto-sklearn requires a Unix-based OS. The approach presented in this study solves the compatibility requirements by the use of docker containers, the provided code contains all necessary configuration files. AutoML is very computationally intensive due to the broad optimization space (feature engineering, models, hyperparameter). A major drawback for the use in the domain of materials design is the need for tabular data to apply the automated workflow shown in Fig. 1. The frameworks do not provide any methods for the featurization of non-tabular data, which is quite common in this domain. In addition, TPOT does not support categorical encoding, so the automated workflow is not applicable to datasets with categorical features. The XAI methods are easy to use for all frameworks. The provided methods are listed in the “Methods” section. MLjar stands out with its out-of-the-box prepared reports and offers the richest XAI functionality. However, for all AutoML frameworks, using ensembles can limit the explainability of the final models.

## Conclusion

In conclusion, a benchmark was set up for four different AutoML frameworks to evaluate twelve datasets from the domain of materials design. Part of the study was a comparison between the manual data mining process in the literature and the AutoML frameworks. The provided scripts allow for an easy transfer of the used methods to additional datasets or AutoML frameworks imposing minimal overhead on the upstream code. The observations prove the following three points: First, modern AutoML frameworks perform slightly better than manually trimmed models on average. This can be achieved with 15 min of training time per data split on a regular grade CPU machine. Second, overfitting is an issue for small datasets in this domain, even for AutoML tools. Third, the sampling strategy highly effects model performance and reproducibility. The implications are not sufficiently considered in most of the evaluated studies. As a result, these findings encourage the use of AutoML in general and special attention on the sampling choice. The latter is especially important for very small datasets for which a nested cross-validation increases the trustworthiness of the results.

The findings and framework from this study can be transferred to other niches. In addition, its impact can be broadened by using a greater variety of tools in the comparison. In terms of usability, an extension to commercial tools for automated machine learning seems promising. Also, the search space of hyperparameters and model types can be extended. Neural architecture search was not included in this study, yet gets a lot of attention in recent years. Lastly, a thorough evaluation of sampling techniques in combination with AutoML frameworks is motivated by the observations on the performance fluctuation.

## Methods

Central task in AutoML frameworks is solving the CASH problem. The four AutoML frameworks applied in this study use different approaches for a solution, described in detail below. Special attention is given to XAI characteristics as H2O and MLjar provide additional functionality in this area.

### Auto-sklearn

The method of Auto-sklearn is described in detail by Feurer et al.^15^ and summarized in the following. Auto-sklearn consists of three major modules: meta-learning, Bayesian optimization (BO) and ensemble building. It is the only framework considered using BO. In order to overcome the slow start of the BO in large hyperparameter spaces, Auto-sklearn offers meta-learning. Meta-learning is intended to persist optimization knowledge between optimizations. Pre-training with BO was performed on 140 datasets from the OpenML repository and the best models as well as dataset characteristics were stored for each task. This serves as an initial knowledge base for further studies. The meta-learning makes use of 38 meta-features, describing dataset characteristics. The distance to the stored datasets in the meta-feature space is calculated when a new dataset is given. The ML models of the n closest datasets (hyperparameter n: initial configurations via metalearning, default n = 25) are the starting points for the following Bayesian optimization of the new dataset. Auto-sklearn uses the model space given by the sklearn library. The Bayesian optimization is data-efficient in finding the best hyperparameters. As a result, it is appropriate for the small datasets considered in this study. However, BO is computationally inefficient for the following reason: all but the best model trained during the optimization are lost in vanilla BO. There is a lot of redundancy as many models performing almost as good as the best model are created. Auto-sklearn utilizes these models by calculating weights for an ensemble selection on a hold-out set. Ensembles can increase performance by leveraging individual strengths of weak learners^61^. In terms of XAI, Auto-sklearn uses the scikit-learn inspection module. This module provides access to partial dependence (PD) plots and individual conditional expectation (ICE) plots, for example.

### H2O

H2O is an AutoML Framework that relies on fast predefined models, random search and stacked ensembles. H2O provides several tools for preprocessing. These include automatic imputation, normalization, feature selection and feature extraction for dimension reduction. The search space for models contains generalized linear models, random forests, extremely randomized trees (Extra-Trees), gradient boosting machines (GBM) and deep neural networks (multi-layer perceptron). Pre-specified models and fixed grid searches are used initially to give a reliable default score for each model mentioned above. A random search within these models is performed in a second iteration. The hyperparameters and their range for the pre-specified models, the grid search and the random search are predefined upon benchmark tests and the experience of expert data scientists. Many models are created within this framework, from which ensembles are built. The ensemble building is done with the training of a meta learner (generalized linear model) to find the optimal combination of the base models. H2O provides an XAI wrapper, which can be applied after the training. It offers PD and ICE plots, learning curves and SHAP-values with the corresponding visualizations.

### MLjar

MLjar provides four predefined working modes (*explain*, *perform*, *compete* and *optuna*) but is also highly customizable. It offers intuitively prepared result explanations out of the box. The mode *compete* was used in this study as it aims to find the best performing ML pipeline. The search space in *compete* contains the following models: linear regression, nearest neighbour, decision tree, random forest, Extra-Trees and GBM (XGBoost, CatBoost, LightGBM). The MLjar framework is defined by four phases. In the first phase, default ML models are optimized with a random search over default predefined hyperparameters. In the second phase, feature construction and selection are performed. So-called golden features are created by mutual combination of all possible unique pairs of features. The feature selection includes original and golden features. A decision tree is trained on these and an additional random feature. The feature importance is determined and all features with lower importance than the random feature are dropped. In the third phase, the fine-tuning of the best performing models is conducted via a random one-factor-at-a-time method. For every model one randomly selected hyperparameter is changed in both directions (higher and lower). In the final phase, all models from the previous steps are used to build an ensemble. The *explain* mode provides a quick and detailed overview on the dataset, by only using default HPO and including all explanation methods. The explanation methods include learning curves, SHAP values and dependency plots as well as a tree-visualization for the tree-based models.

**Table 4:** Availability of the datasets.

Alias	Availability	open access
Guo-2019	data mendeley^36^	✗
UCI-concrete	UCI data repository (Link 1)	$\checkmark$
Yin-2021	GitHub (Link 2)	$\checkmark$
Hu-2021	upon reasonable request	✗
Matbench-steels	data repository (Link 3)	$\checkmark$
Atici-2011	Paper^31^	✗
Su-2020-2	Paper^41^	✗
Su-2020-1	Paper^41^	✗
Huang-2021	Paper^33^	✗
Bachir-2018	Paper^32^	$\checkmark$
Koya-2018	Paper^34^	$\checkmark$
Xiong-2014	Paper^39^	$\checkmark$

Link 1: https://archive.ics.uci.edu/ml/datasets/concrete+compressive+strength.

Link 2: https://github.com/Binbin202/ML-Data.

Link 3: https://matbench.materialsproject.org.

### TPOT

The tree-based pipeline optimization tool (TPOT) is an AutoML framework, which is based on an evolutionary algorithm using genetic programming^17^. TPOT build flexible tree-based pipelines from a series of pipeline operators. These operators are various data transformation operators or ML models from the sklearn library. The root of every tree-based pipeline starts with one or several copies of the input data. The input data is fed into the different available pipeline operators. Every pipeline operator modifies the provided data and then passes the resulting data further up the tree. The resulting prediction of the TPOT-pipeline is made when the data is passed through the final ML model. TPOT provides a wide range of preprocessing options. Among those are feature construction, feature selection, feature transformation and feature combination, but the handling of categorical features is not included. The structure of the tree-based pipeline and the hyperparameters of the single operators are optimized via genetic programming. A fixed number (population size) of tree-based pipelines is generated at the beginning of the optimization. These pipelines represent the initial generation. They are evaluated through the optimization criteria, i.e., the regression or classification score. The score is used to select pipelines, which are then randomly changed by a broad set of modifications to create the next generation. Additionally, the best pipelines of the old generation are transferred to the next generation. This process is repeated for a user-defined time or number of generations. TPOT offers no further functionality for XAI aside from feature importance.

## Data Availability

The datasets for the presented study are open access or available from the corresponding author on reasonable request, see Table 4 for information on the individual datasets.

## Code availability

The experimental setup is available on GitHub: https://github.com/mm-tud/automl-materials.
